# Is the Prejudice Towards People With Borderline Personality Disorder Model Reflective of Lived Experience?

**DOI:** 10.1111/inm.70258

**Published:** 2026-04-17

**Authors:** Hannah Sheppard, Elizabeth Huxley, Boris Bizumic, Alison L. Calear

**Affiliations:** ^1^ School of Medicine and Psychology Australian National University Canberra Australia; ^2^ Centre for Mental Health Research Australian National University Canberra Australia

**Keywords:** borderline personality disorder, lived experience, prejudice, prejudice model, stigma

## Abstract

The high levels of stigma faced by people with borderline personality disorder (BPD) is well recognized but inconsistently operationalized within the literature through the use of multiple instruments that measure different prejudicial attitudes. The Prejudice towards People with Borderline Personality Disorder (PPBPD) framework was developed to provide a comprehensive model and psychometrically sound scale for measuring prejudice. The four PPBPD subscales encompass the different prejudicial attitudes measured across other existing instruments, namely Fear/Avoidance, Malevolence, Authoritarianism, and Unpredictability. The PPBPD framework, however, was developed in isolation of the lived experience. To address this, we conducted online interviews with 13 Australians with BPD to investigate if the PPBPD's four‐factor model reflects the attitudes they have experienced. The interviews discussed participants' experiences of support and prejudice and their impacts, the PPBPD model, and help‐seeking. Participants felt the model was an accurate representation of the prejudice they had experienced, and all participants identified experiences that related to each of the four dimensions. Important sources and impacts of each dimension of prejudice were also identified. The results suggest that the PPBPD model and scale are useful tools for measuring and understanding prejudice towards people with BPD and contextualizing it within lived experiences. Consulting people who have BPD is a key step in providing evidence for the usefulness of the PPBPD model and scale. Using the PPBPD model can provide a more comprehensive understanding of the attitudes underlying the stigma faced by people with BPD and support the development of appropriate stigma reduction interventions.

## 1 Background

Although various approaches to conceptualizing borderline personality disorder (BPD) exist (e.g., the ICD‐11, World Health Organization 2021; or the DSM‐5 models, American Psychiatric Association 2022), the core dimensions of the disorder are widely agreed to be interpersonal hypersensitivity, emotional dysregulation, and impulsivity and behavioural dysregulation (Trull and Hepp 2023). The lifetime prevalence of BPD is estimated to be 0.7%–2.7% in the general adult population, 12% in people accessing outpatient clinics, and 22% in those accessing inpatient clinics (for a review, see Leichsenring et al. 2024). People with BPD face high levels of stigma, which is primarily ascribed to a lack of knowledge of BPD, misattributing the symptoms as intentional “misbehaviour”, and perceptions that BPD is difficult or impossible to treat (for a review, see Ociskova et al. 2023).

People living with BPD have consistently reported that not being taken seriously, discrimination, and lack of respect are three of the greatest concerns when attempting to seek help from services (Lawn et al. 2017). In 2018, Australian mental health consumers and carers identified stigma around BPD as a research priority (Banfield et al. 2018). Corrigan and Kosyluk's (2014) social‐cognitive model views stigma as a process, where endorsed stereotypes result in negative attitudes (or prejudice), which in turn lead to discrimination. Following Eagly and Chaiken (2007), we define an attitude as an evaluative cognitive, affective, or behavioural response to a specific entity. As stereotypes are commonly pervasive and resistant to change (Corrigan and Penn 2015), whereas attitudes are often malleable (Bizumic and Sheppard 2024) and mediate between stereotypes and discrimination, prejudice presents the most promising pathway to reducing stigma (Kenny et al. 2018). However, understanding and addressing prejudice towards people with BPD is hindered by the use of multiple instruments that often measure different attitudes (e.g., fear or callousness) and have limited empirical support (Baker and Beazley 2022). Consistent utilization of a psychometrically sound instrument across studies would greatly enhance research efforts.

The Prejudice towards People with BPD (PPBPD) model provides a comprehensive, well‐validated measure of prejudice towards people with BPD (Sheppard et al. 2023). The four‐factor model comprises Fear/Avoidance (a desire for social distance from, difficulty interacting with, and feeling unsafe around people who have BPD), Malevolence (a lack of sympathy for, resentment towards, and beliefs of inferiority about people who have BPD), Authoritarianism (controlling and restricting choices of people with BPD), and Unpredictability (viewing people who have BPD as completely unpredictable). Each factor is bipolar, enabling the scale to assess both support for and prejudice towards people with BPD.

The PPBPD model was adapted from the Prejudice towards People with Mental Illness (PPMI) model, which was developed from the broader mental health stigma literature (Kenny et al. 2018). The four PPBPD subscales encompass attitudes that are measured across other existing prejudice instruments (e.g., Black et al. 2011; Shanks et al. 2011) and empirical evidence for the PPMI scale has been reported across multiple studies (e.g., Bizumic et al. 2022; Bizumic and Gunningham 2022; Kenny et al. 2018; Sander et al. 2022). Nonetheless, the model was developed without input from people with BPD. Research suggests that people with BPD face high levels of stigma, in that people with BPD may be framed as having a behavioural issue, rather than a mental health condition, mislabelled as “manipulative” or “attention‐seeking”, and are met with less sympathy and optimism, less belief in their need for professional help, and greater desires for social distance than people with other common mental health conditions (e.g., Bodner et al. 2015; Elliott and Ragsdale 2024; Furnham et al. 2015; Sheehan et al. 2016; Sulzer 2015). Thus, it is important to investigate whether the four‐factor PPBPD model comprehensively captures the prejudice experienced by people with BPD, or whether the differences in reported stigma are indicative of a BPD‐specific dimension of prejudice that is not included within the PPBPD model.

## 2 Aims

The central aim of the current study was to examine, through semi‐structured interviews, whether the four‐factor PPBPD model was reflective of the prejudice faced by people with BPD, or if additional factors more specific to the experiences of people living with BPD were missing from the model. The secondary aim was to contextualize the PPBPD model within the lived experience by identifying sources and impacts of prejudice and potential avenues of prejudice reduction.

## 3 Methods

### 3.1 Ethical Considerations

This study was reviewed and approved by the Australian National University Human Research Ethics Committee (Protocol: 2021/397), and the procedures followed were in accordance with the Helsinki Declaration as revised in 2013. The participants provided their written informed consent to participate in this study. Participants also provided informed oral consent before commencing the interview.

Prior to expressing interest, participants were provided with an information sheet that explained the content and expected benefit of the interviews, as well as their voluntary nature. Participants had access to the questions prior to the interview and were able to skip any questions that they felt uncomfortable answering. Support resources were provided in the information sheet and again after the interview. The interviewer, a PhD candidate, was trained in mental health distress and risk management, and a distress protocol developed by the ACT Consumer and Carer Mental Health Research Unit (2016) was followed if a participant showed signs of distress (Calear et al. 2024, appendix 20.1). As part of this protocol, a registered clinical psychologist was available in case a participant became distressed; however, they were not required for any interview. A debrief with the psychologist was also offered to all participants upon completion of the interview, though no participant chose to do so.

### 3.2 Design

A generic qualitative approach was considered appropriate for this study as the aims focused on the content of the interviewees' opinions, attitudes, and reflections on their experiences with prejudice towards people with BPD. Generic qualitative inquiry is recommended when the research aims to investigate the experiences of a phenomenon and describe pre‐existing knowledge about the topic (in this case the four dimensions of prejudice) from the perspective of the participant (Percy et al. 2015).

### 3.3 Participants

Australians who had been diagnosed with BPD and were comfortable discussing experiences with prejudice were sought for this study. Participants self‐reported their BPD diagnosis and how long it had been (in years) since they had received the diagnosis. To be eligible for the study, participants also needed to be 18 or older and confirm that they had a support person they could contact if needed post‐interview. Having comorbid conditions did not exclude potential participants, as comorbidities with BPD are common (Tate et al. 2022), and excluding participants with comorbidities would not provide a comprehensive sample. Twelve participants (92.3%) disclosed comorbidities. The experiences participants shared in the interviews demonstrated that they were able to distinguish between perceived attitudes towards their mental health in general and attitudes towards BPD specifically.

A convenience sample of 13 participants was recruited online via a national BPD organization. The sample size is consistent with qualitative research guidelines (Hennink and Kaiser 2022) and supported by the specificity of the sample, the focused aim and use of established theory, and the depth of information shared by the interviewees (Malterud et al. 2016).

All 13 participants identified as Australian and spoke English (see Table 1). Nine (69.2%) identified as female, two (15.4%) as male, and two (15.4%) as non‐binary. Most participants were Anglo/Caucasian/White (*n* = 11, 84.6%) and reported a lower‐than‐average socio‐economic status (*n* = 6, 46.2%). Participant age ranged from 25 to 65 years, with the most common age bracket being 25–34 years (*n* = 4, 30.8%). Quotes presented throughout the results were chosen such that each interviewee was represented. As a result, the majority of quotes presented are from female participants (*n* = 21), with quotes from non‐binary (*n* = 5) and male (*n* = 4) participants reflecting the smaller number of non‐female participants in the sample.

**TABLE 1:** Summary of participant demographics.

Category	Sub‐category	*N*
Gender	Female	9
	Male	2
	Non binary	2
Age	25–34	4
	35–44	3
	45–54	3
	55–64	2
	Not stated	1
Socioeconomic status	Much lower than average	5
	Lower than average	6
	Average	1
	Higher than average	1
Ethnicity	Anglo/Caucasian/White	11
	Other/not stated	2

### 3.4 Data Collection

Online, semi‐structured interviews were conducted via Zoom by the first author from October 2022 to January 2023. Prior to the interviews, the interviewer was not known to any of the participants. Participants were permitted to have other people present during their interview; however, none chose to do so. The interviews ran for an average of 90 min, and participants were compensated with a $AUD 50 gift card for their participation.

**FIGURE 1:**
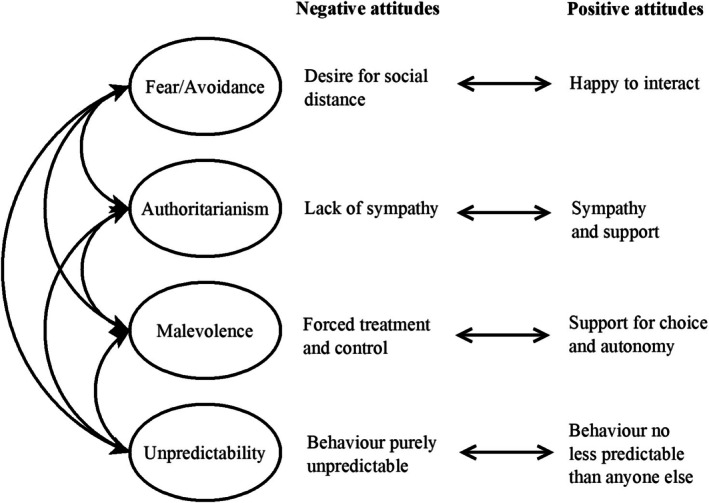
The diagram used when discussing the PPBPD model with participants.

Based on the PPBPD model and research aims, a bespoke interview guide was developed by the research team, consisting of a PhD candidate and three senior academics who had a range of expertise spanning qualitative methods, clinical psychology, public health, and mental health consumer engagement (see Supporting Information S1). Each interview followed the interview guide, which began with open‐ended questions relating to how participants felt about themselves and others with BPD, how they believed people without BPD perceived them, and if they had experienced prejudice related to their having BPD. Participants were then asked if they had experienced prejudice or support that aligned with the four PPBPD factors. Next, participants were introduced to the PPBPD model, discussed it, and provided feedback (see Figure 1 for the illustration used in these discussions). Finally, the interviews finished with open‐ended questions related to help‐seeking and stigma reduction.

The first author transcribed the interviews verbatim, which allowed for familiarity with and constancy across transcriptions. Audio recordings and transcripts of the interviews were stored on a password‐protected laptop accessible only to the first author. Once transcription was complete, the recordings were deleted. Any identifying information was removed from the transcripts, and pseudonyms were assigned to each participant and used in the analysis. Transcripts were returned to each participant to edit, provide input, or withdraw it from the analysis (no participant chose to withdraw). Anonymised transcripts were accessible to the members of the research team for analysis.

Approaches to reflexivity, such as that of Lazard and McAvoy (2020), guided reflection and evaluation of the assumptions, positionality, and affective responses of the research team and their influence throughout the research process to mitigate bias and possible power dynamics between the interviewer and participants, where possible. After each interview, the interviewer engaged in written reflection and debriefing discussions to reflect on the content of the interview and how it related to her assumptions and the answers shared by previous interviewees. Reflection on how the interviewer's perspectives, assumptions, and goals changed continued throughout the study. Regular discussions with the other members of the research team promoted the sharing of differing perspectives and the resolution of disagreements in interpretations of themes and codes identified during the analysis.

### 3.5 Analysis

**FIGURE 2:**
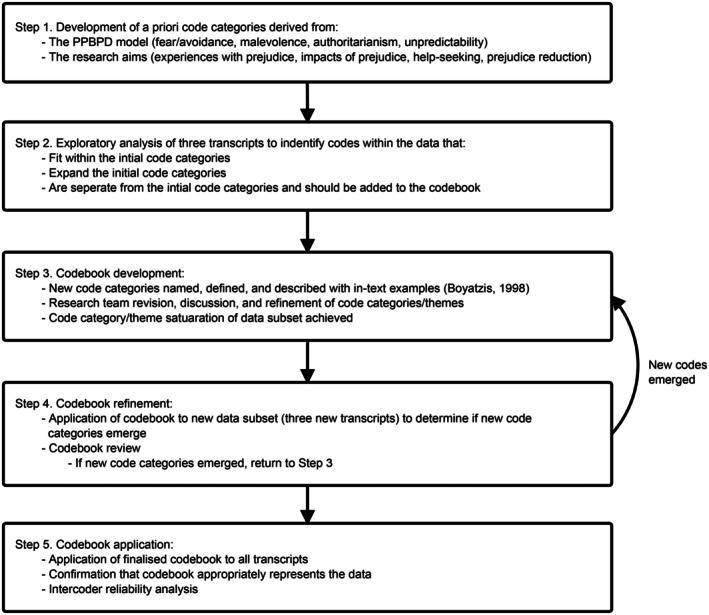
Codebook creation and application process.

Using NVivo 14, theoretical analysis was employed (Percy et al. 2015) guided by the development of a codebook (Fereday and Muir‐Cochrane 2006; Roberts et al. 2019; see Figure 2). Theoretical thematic analysis is driven by theory and predetermined themes, while also allowing for new patterns (or code categories) and themes to emerge from the data (Percy et al. 2015). An a priori codebook based on the PPBPD model (fear/avoidance, malevolence, authoritarianism, and unpredictability) and research aims (experiences with prejudice, impacts of prejudice, help‐seeking, and prejudice reduction) was developed to guide the thematic analysis.

Each transcript was read multiple times by the first author and segments of text that appeared meaningful were highlighted (Percy et al. 2015). The highlighted segments (codes) were then compared to the a priori codebook to determine if they fit within, expanded upon, or were separate from the existing code categories (Roberts et al. 2019). All code categories were given a name and definition, and they were illustrated with in‐text examples (Boyatzis 1998). The codebook was reviewed and refined by the research team, and transcripts were reread by the first author until no new code categories emerged. Once all transcripts had been analyzed and no new codes emerged, the codebook was considered an accurate representation of the data (see Table 2).

**TABLE 2:** The themes and sub‐themes as they were defined in the codebook.

Theme	Definition	Interview example
**Experiences with prejudice**	Described experiences or perceptions of prejudice towards someone/people who have BPD.	“They don't believe me generally. And they don't believe that I have a disability, and they see me functioning reasonably well on the outside.” (Drew)
**Fear/Avoidance**		
Fear/Avoidance Present	Experiences or attitudes related to beliefs that people with BPD should be avoided and rejected from the community, that they are difficult to interact with, and/or dangerous. Additional coding: source present/absent	“I would tell people that I have BPD, and I have seen people cross their arms turn away from me and move away from me. People in the mental health field.” (Drew)
Fear/Avoidance Absent	Experiences or attitudes related to beliefs that people with BPD should be accepted and integrated into the community, that they are easy to interact with, and/or that they are harmless. Additional coding: source present/absent	“But I've had people who stuck around through the worst, when my symptoms were at their worst…” (Emma)
**Malevolence**		
Malevolence Present	Experiences or attitudes related to resentment towards people with BPD, or beliefs that people with BPD do not deserve sympathy or support and/or are inferior to other people. Additional coding: source present/absent	“Very much the attitude has been that ‘[people with BPD] are just spoiled brats and we're just pampering them and catering to them and giving them handouts. And they're just manipulating the system’.” (Alice)
Malevolence Absent	Experiences or attitudes related to beliefs that people with BPD deserve sympathy and support, and/or are the same as other people. Additional coding: source present/absent	“Everybody can be diagnosed with something by surprise… You're not superior just because you don't have a diagnosis.” (Alex)
**Authoritarianism**		
Authoritarianism Present	Experiences or attitudes related to beliefs that people with BPD should be controlled by others by any means necessary. Additional coding: source present/absent	“…it's [being] ignored, and also just the lack of, like, respect and autonomy.” (Emma, things that stop her help‐seeking)
Authoritarianism Absent	Experiences or attitudes related to supporting people with BPD being free to control their own lives. Additional coding: source present/absent	“Choice is so important. It's a shame people can't see the value of having treatment… But you can't force something on somebody.” (Talia)
**Unpredictability**		
Unpredictability Present	Experiences related to considering all people with BPD behave unpredictably, that people with BPD are more unpredictable than people without BPD. Additional coding: source present/absent	“With my husband, I think he feels I can be unpredictable at home. I know that my daughter feels that way.” (Mary)
Unpredictability Absent	Experiences related to beliefs that people with BPD behave predictability. Additional coding: source present/absent	“I think they're very predictable if you know their story.” (Chloe)
**Impacts of prejudice**	The impacts that prejudice towards people with BPD had on the interviewees.	
Self	Experiences of feeling dismissed, invalidated, or rejected when trying to share feelings and experiences or seek care and support, feeling isolated, expressions of feeling a lack of hope for the future or treatment, loss of trust in others, negative self‐image, feelings of shame related to having BP or expressions of prolonged negative affect as a result of prejudicial experiences.	“My self‐esteem has gone down significantly.” (Emily)
Social	Impacts on social experiences, such as estrangement from family, friendships ending or being strained, ceasing community involvement.	“I over analyze everything about myself still because I don't want to upset somebody else…” (Alex, when discussing experiences related to being considered unpredictable)
Employment	Impacts on ability to work or workplace environment (feeling comfortable, safe, judged, supported).	“I strongly suspect that I'm facing prejudice when applying for work.” (Mary)
Future help‐seeking	Expressions of being less likely to seek help in the future due to past experiences with prejudice.	“Yeah, because I know they say one thing and then they behave another way.” (Emily, when asked if the stigma around BPD stops her seeking help)
Treatment of other conditions	Expressions of being less likely to seek help for, or having been unable access care/treatment for, other physical or mental health conditions due to others prejudice towards them having BPD.	“They just see my diagnosis and say, ‘oh, she's got BPD. So, we'll just let her sleep it off and send her home in a few hours, or once you settle down’ … I've tried to explain to them that they are separate diagnoses.” (Hazel, discussing trying to seek help for symptoms unrelated to BPD)
**Help‐seeking experiences**	Experiences related to help‐seeking including when or why the interviewee sought care, which services they sought out, barriers they experienced, and experiences seeking or receiving a diagnosis. Additional coding: positive/negative.	“[Carers and physicians] were more often than not caring and understanding. And if they didn't understand they would refer me to somebody else. But they never judged me.” (Daniel)
BPD faces more prejudice	Beliefs that BPD faces different types or higher levels of prejudice than other diagnosis groups.	
Direct expression	Direct expressions that BPD faces higher or different levels of prejudice.	“I feel like there's less help and less willingness to help. There's much more onus on the individual to sort their shit out.” (Olivia)
Indirect expression	Statements or behaviours that indicate an expectation that people with BPD face higher levels of prejudice, such as being willing to disclose another mental health diagnosis, but not a BPD diagnosis.	“I might say I have mental health conditions, but I would say like bipolar because it has less of a stigma attached to it.” (Phillipa)
BPD literacy		
The term borderline personality disorder	Believing that the name borderline personality disorder is not a good representation of the symptoms or increases stigma or confusion around the diagnostic criteria.	“It is that stigma of BPD, and personality disorders, they misbelieve that and it must mean that you have more than two personalities…” (Phillipa)
General lack of knowledge	Having no knowledge or incorrect knowledge about BPD. Additional coding: source present/absent.	“I think the prejudice comes because people just don't understand you… And so, they tend to back away in relationships.” (Jacob)
How to care for/treat someone who has BPD	Having no knowledge or incorrect knowledge of BPD treatment and support options. This includes not knowing how or where to refer a patient with BPD to appropriate treatment/support pathways.	“I don't think they understand what they're treating at all. I think they just see the keywords… So, I think they miss a lot of the finer points.” (Daniel)
**Prejudice reduction**	The interviewees thoughts on prejudice reduction, including where they think efforts need to be focused, where they should be focused first, what they feel could be/have found to be helpful, and what it would mean to them if prejudice towards people with BPD was reduced.	“Potentially, looking at kind of those large generalizations, particularly the ‘manipulation’. I think for healthcare professionals that's one that really needs to be addressed.” (Chloe)
Additional coding		
Source of attitudes/experience	If present, then coded: community, family, friends, government services, healthcare providers, partner, self, “they”, or workplace If absent or otherwise unclear, then coded: not identified	
Positive/negative experience	If overall positive experience, then coded: positive If overall negative experience, then coded: negative	

*Note:* Bold text indicates themes that were pre‐determined from the PPBPD model and the research aims. All other code categories emerged from patterns/clusters of codes during the analysis.

The first author used the final codebook to code all transcripts. The themes of prejudice discussed by participants were explored in two ways. First, participant responses to the first six general, open‐ended questions were analyzed to explore their perceptions of how people with BPD are viewed and their general experiences with prejudice. This was done to ensure themes were driven by participant responses, not the questions asked regarding the model. Second, the codebook was applied to the entire transcript to include participant responses to all interview questions. A second researcher independently coded 10% of the data for inter‐rater reliability analysis. Cohen's kappa coefficient was calculated as κ = 0.91, showing a high level of agreement between coders (Viera and Garrett 2005).

## 4 Results

### 4.1 Perceptions of People With BPD and Experiences With Prejudice

In response to the initial general, open‐ended questions, all 13 participants shared experiences with prejudice. Participants discussed prejudice in a broader context as general messages that they had received or an amalgamation of many experiences. When asked how she thought other people perceived people with BPD, Olivia responded “As dysregulated, as they overreact, attention seeking, unable to manage themselves. Negative. Like there's so many things but negatively, overall, negatively.”

All participants shared experiences with specific situations and sources, such as friends, family, and community groups. Many participants shared experiences of prejudice from healthcare providers (HCPs).

Mostly in hospital settings. When I was really unwell, I had a lot of ED presentations. Psychiatric presentations. And I felt very dismissed. And I think that was because I was displaying high level BPD traits. So, the very first instance that I presented for suicidal ideation, I was pretty much given an AA pamphlet and told to go home. So that's very traumatic. (Emma)

Thematic grouping of these experiences found all four dimensions of prejudice. Participants identified attitudes related to desires for social distance, fear, and discomfort interacting with people who have BPD, which all reflect the fear/avoidance dimension of prejudice. Other people perceiving the behaviour and emotions of people living with BPD as completely unpredictable, out of control, or reactive were discussed by six participants. One participant also shared an experience with authoritarianism attitudes, where other people restricted the participant's involvement in a community space due to them having BPD. Attitudes related to malevolence were most prevalent, as almost all participants discussed feeling blamed for their condition or symptom presentation, callousness from others, and perceptions that the participants were manipulative or attention‐seeking rather than unwell. For example, when discussing the prejudice she had experienced, Alice said “that ‘you're just a spoilt, manipulative drama queen’. ‘You're just looking for attention’. ‘There's nothing really wrong with you’. Yeah, that's probably the main thing, ‘There's nothing actually wrong with you’”.

### 4.2 Experiences With the PPBPD Dimensions

Throughout each interview, all participants described experiences with fear/avoidance attitudes. Incidences where others withdrew from friendships with the participant due to their having BPD were common. Participants shared how friends, family, HCPs, or acquaintances would express discomfort or nervousness when interacting with them and reduce or cease communication. Others shared how they were excluded from social events by friends, family, or community groups. Attitudes reflecting an absence of fear/avoidance prejudice were also evident, as participants shared how friends and family stuck by them, actively involved them in social events, and treated them the same as they had before their diagnosis was disclosed. Others shared how community members and acquaintances were open to discussing BPD and sharing experiences, happy to initiate friendships, and treated them the same as everybody else.

All participants shared experiences that aligned with the malevolence dimension. These attitudes were characterized by others communicating that people with BPD are manipulative, bad, or attention‐seeking, not trying to get better, or are undeserving of sympathy, support, or care. Nonetheless, the absence of malevolence was also highlighted, as all participants shared how some family, friends, HCPs, colleagues, and acquaintances could be caring and sympathetic through efforts to understand, improve knowledge of BPD, and provide support. Others expressing the desire or actively attempting to improve things, such as access to care and support and overall acceptance of people living with BPD, were also discussed.

Attitudes that reflected authoritarianism were the least prevalent across the interviews. Instances of authoritarianism were characterized as having little or no choice and autonomy in treatment, being forced into treatment, pushed to stay in treatment, and others not allowing choices in their personal life. Participants shared how these attitudes came from people who wanted to help, such as HCPs who were sure the treatment would help, or family who pressured them into continuing to see a HCP when the participant felt it was unnecessary. However, many participants felt that treatment was not helpful if the person was not ready to receive it. Conversely, participants discussed experiences with people who supported their choice and autonomy in treatment and life decisions, as well as how open discussion about and waiting for the right time to make life decisions was more beneficial in the long run. Most attitudes reflecting an absence of authoritarianism came from the participants themselves.

Last, all participants shared how people with BPD, and their behaviours and emotions, are perceived as out of control or completely unpredictable. They felt that other people judged their overall behaviour based on moments when they were dysregulated or expected them to behave in unpredictable ways after hearing about their BPD diagnosis. For example, Alice shared how people's behaviour changed after she told them that she has BPD, “…they treated me differently as though they had to walk on eggshells”. Three participants shared personal attitudes reflecting the absence of unpredictability attitudes, which were all characterized by believing that people with BPD are individuals with their own story that informed predictable behaviour patterns.

### 4.3 Direct Discussions of the PPBPD Model

Throughout the discussion of the PPBPD model, 12 participants were able to relate their experiences to the model, particularly the bipolar nature of the factors: “I personally identify with that because you experience, there's sympathy and there's support, but they will still look out for unpredictable behaviour.” (Emily). Generally, the participants felt that the four factors were an accurate reflection of their experiences. For example, when discussing Figure 1, Alex said “With regards to BPD, I think that's probably the core ones that I could think of, like everything that I'm thinking of would fit into one of these categories somewhere on the left or the right.” One participant indicated that they did not think they had enough overall experience with prejudice to provide input on the model. They discussed how a model akin to the PPBPD model could be helpful in identifying antecedents of prejudice and reducing discrimination, particularly fear/avoidance, but were unable to provide input on the accuracy of the four factors. Nevertheless, when discussing their experiences of both support and prejudice, this participant shared experiences that reflected all four dimensions.

Two participants discussed possible improvements for the PPBPD model. Initially, Alex was unsure about the malevolence factor and felt that empathy (rather than sympathy) may be a better representation of the prejudice they had experienced, stating “I don't necessarily know that sympathy is my favourite term for that”. However, after further reflection (without interviewer input) they decided that sympathy was an acceptable descriptor of the factor. Additionally, Emma was unsure if the model represented the lack of hope that she had experienced from HCPs (“this sort of attitude of ‘Oh, it's too hard to treat. They won't put in the work. The outcome is really poor’”) and felt that “it almost fits under malevolence, but not quite”.

### 4.4 Contextualizing the Model

Participants highlighted four associated themes that related to their experiences of prejudice and provided important information for understanding how the PPBPD dimensions are experienced.

#### 4.4.1 Sources of Attitudes

**FIGURE 3:**
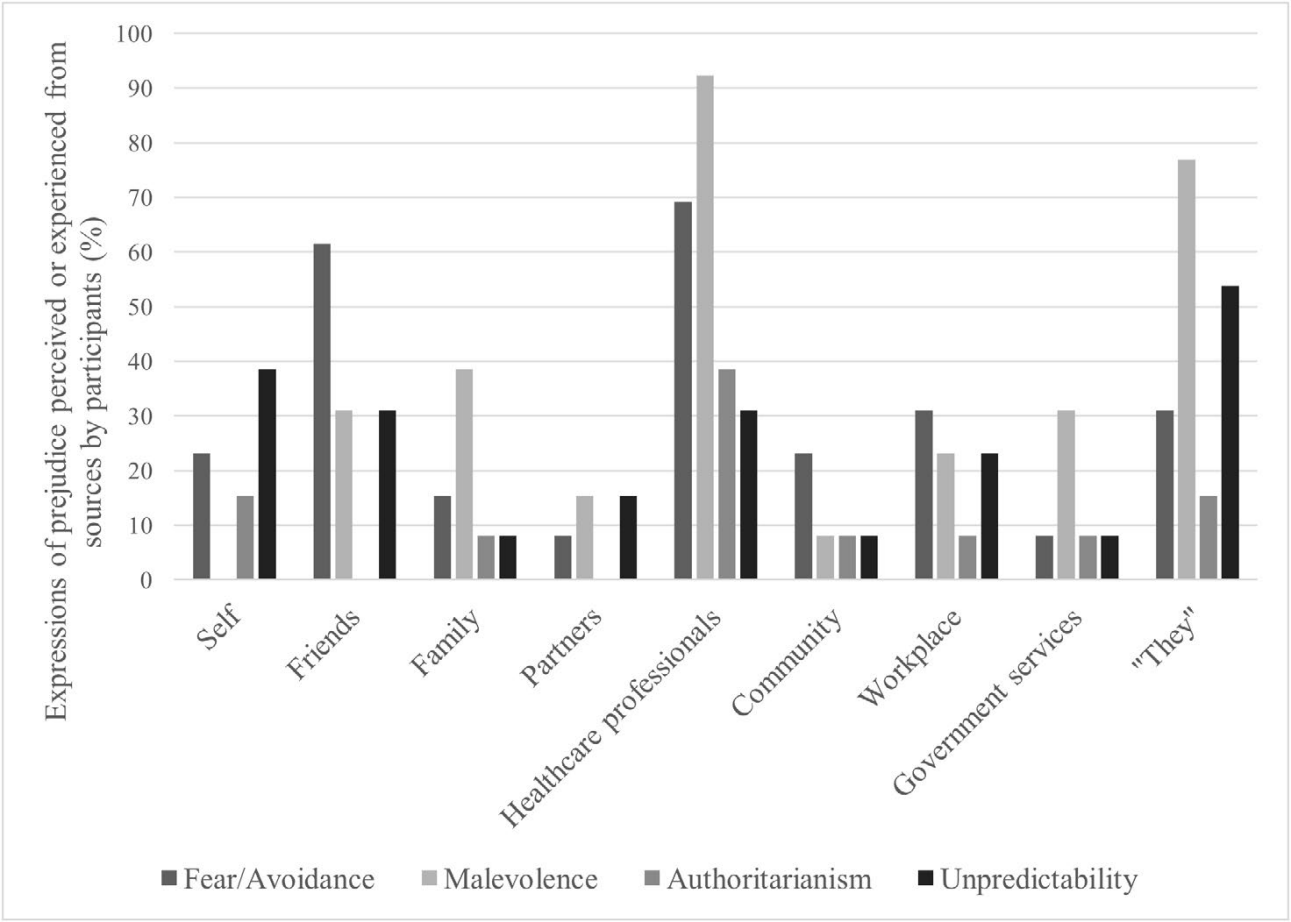
Sources of prejudice.

The experiences of both prejudice and support shared by participants emphasized 10 sources: the participants themselves, friends, family, partners, HCPs, the broader community (e.g., church groups), workplaces, support groups (e.g., non‐government organizations), government services (e.g., the police or national disability services), and a broader “they” category (e.g., “they”, “people”, or “others”). Figure 3 presents the percentage of participants who discussed each dimension in relation to a specific source. Overall, attitudes or experiences related to HCPs were most commonly shared, with 12 of the 13 participants (92.3%) discussing experiences or perceptions of malevolence involving HCPs.

Attitudes reflecting an absence of fear/avoidance prejudice were most evident when participants discussed experiences with friends. For example, Jacob discussed how good friends would listen to him explain BPD and his experiences and respond with a willingness to work with him in their friendship. An absence of malevolence prejudice was most common in experiences with BPD support groups. For instance, Mary shared how the local BPD support group was one of her greatest sources of acceptance and support. HCPs were identified as the main source of attitudes reflecting the absence of authoritarianism prejudice, as some participants discussed how their HCPs would encourage discussion and participant input in treatment options. The absence of unpredictability prejudice only came from the participants themselves. Across all dimensions, participants identified themselves as a source of low prejudice. For example, Phillipa shared, “I think people with BPD are actually pretty amazing people, particularly if they have done their best to try and stay alive.”

#### 4.4.2 Impacts of Prejudice

**FIGURE 4:**
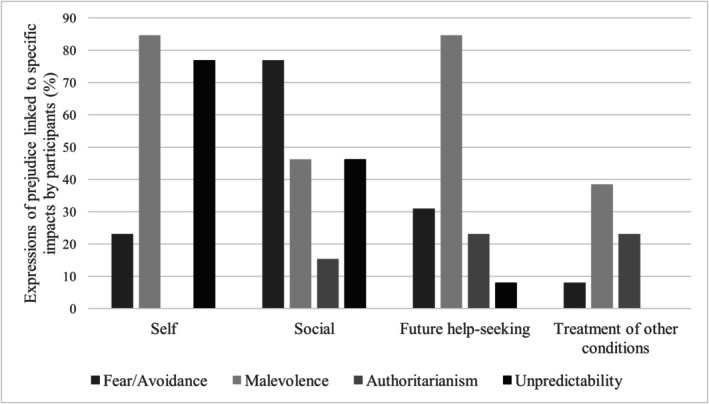
Impacts of prejudice.

All participants discussed how prejudice impacted multiple aspects of their lives, highlighting five subthemes: self, social, employment, future help‐seeking, and treatment of other conditions. Figure 4 presents the percentage of participants who discussed each of the impact subthemes in relation to each PPBPD factor. The impacts on employment are not included in the figure because this subtheme was more widely attributed to prejudice as a whole, rather than a specific factor.

Participants described how their experiences with prejudice had impacted them as individuals. These impacts were characterized by isolation, negative self‐image, prolonged negative affect, lack of hope for the future, and shame. The impacts of prejudice on social relationships were also widely shared and comprised being excluded from social events and groups, difficulties initiating and maintaining relationships, reduced trust in others, and lack of acceptance or estrangement from family. The impacts of prejudice extended into the workplace, where prejudice created challenges gaining and maintaining employment or impacted their relationships with colleagues and treatment within the workplace.

Participants described how general perceptions of people with BPD and their behaviours, as well as past negative experiences with help‐seeking, diminished their trust in healthcare services. These experiences reduced almost all participants' likelihood of seeking further support or treatment for their BPD symptoms. Similarly, some participants shared how the prejudice they experienced towards their having BPD impacted their likelihood or ability to seek care for other conditions, such as having concerns of other mental and physical health conditions being dismissed or overlooked.

#### 4.4.3 A BPD Diagnosis Faces More Prejudice

Many participants felt that people with BPD received more prejudice than people with other mental health conditions. Some participants directly expressed this belief or shared experiences where people with BPD faced less favourable attitudes than other diagnosis groups. These instances were most commonly associated with attitudes that reflect malevolence prejudice, as participants discussed receiving less sympathy or compassion towards their BPD and being less likely to be considered truly ill when seeking help. For others this was expressed indirectly through discussing their willingness to disclose one mental health condition (e.g., bipolar) but not disclose having BPD.

#### 4.4.4 BPD Literacy

Eleven participants discussed concerns with low BPD literacy, and most frequently associated with it fear/avoidance prejudice. Participants discussed how access to misinformation, non‐lived experience‐based information, or a general lack of knowledge about BPD led to misunderstandings and social distance. When seeking help, some participants experienced HCPs who did not understand how to provide care or treatment for someone who has BPD, often resulting in poor or no treatment. Concerns with the term *borderline personality disorder* were also raised. In particular, some participants felt that the term *personality disorder* may mislead people to believe they had innate problems with their personality rather than a treatable disorder.

## 5 Discussion

This study sought to determine if the PPBPD model accurately reflected the experiences of people living with BPD and to identify links between the four factors of prejudice and the sources and impacts. Through both direct and indirect means, the model was supported by the interview participants. Both positive and negative experiences shared by the participants related to one or more of the four dimensions of prejudice. These shared experiences provided understanding of how the PPBPD model is characterized and experienced by the participants. This suggests that factors of Fear/Avoidance, Malevolence, Authoritarianism, and Unpredictability found within the PPBPD model represent aspects of the lived experiences of support for and prejudice towards people with BPD. Consequently, this indicated that the PPBPD model and associated scale are useful tools for not only measuring prejudice but organizing and integrating prejudice research, and thus deepening our understanding of prejudice towards people with BPD.

Two concerns about the PPBPD model were raised that need to be considered. First, one participant raised a concern regarding the focus on sympathy rather than empathy in the malevolence factor. The distinction between sympathy and empathy lies in the orientation of the feelings, where empathy reflects a shared feeling *with* another person and sympathy denotes feeling *for* another person (Singer and Lamm 2009). It is the orientation towards the other person that lends sympathy to be encompassed within the PPBPD model, as prejudice involves a negative attitude *towards* a group or person (Allport 1954). They are related constructs; however, with empathy showing a consistent relationship with prejudice across research (McFarland 2010), particularly the Malevolence subscale (Bizumic and Gunningham 2022; Kenny et al. 2018; Sheppard et al. 2023, 2025). Alex's preference for empathy over sympathy is reflected in the literature (for a discussion, see Gerace 2020). Coupled with the prevalence of malevolence identified throughout the interviews, Alex's perspective highlights the need for further investigation of the malevolence factor to explore how constructs like sympathy, empathy, and compassion fit within or relate to the PPBPD model and ensure that the correct terminology is utilized.

Second, another participant felt that a lack of hope for her future and recovery from HCPs was not adequately addressed in the PPBPD model and should be further investigated. Prior research has also identified belief in the patient's ability to recover as an important aspect of positive therapeutic relationships for people with BPD (Romeu‐Labayen et al. 2020). Sheppard et al. (2025) investigated how belief in treatment effectiveness and patient recovery related to the PPBPD model in a sample of 593 HCPs. They reported that belief in patient recovery demonstrated a significant correlation with the PPBPD scale (*r* = −0.50, *p* < 0.001), specifically with the Fear/Avoidance subscale (semipartial *r* = −0.34, *p* < 0.001). Emma's observation supports the need for further research into this association, as well as intervention development, where interventions may highlight success stories from both people with BPD and HCPs in the hopes of increasing belief in patient recovery and reducing prejudice.

Analysis of the interviews indicated four associated themes: “sources of attitudes”, “impacts of prejudice”, “a BPD diagnosis faces more prejudice”, and “BPD literacy”. The associated themes are not novel to the literature, as higher levels of stigma and low BPD literacy have been pervasive issues in BPD stigma research (for a review, see Ring and Lawn 2019). Nevertheless, connecting those associated themes to the four dimensions of prejudice provides tools for deepening understanding of stigma and how the negative attitudes might be reduced. For example, knowledge of the biogenetic causes of mental health conditions has been found to correlate with increased desire for social distance and beliefs of dangerousness (Kvaale et al. 2013). Another study found that teaching psychology students about dimensional personality disorder models (such as the alternative model for personality disorders, American Psychiatric Association 2013) indirectly reduced prejudice, particularly fear/avoidance, through increased continuum beliefs, decreased categorical beliefs, and perceived ingroup membership (Wai et al. 2024). These findings indicate that particular types of education may be more effective, or potentially harmful, when reducing fear/avoidance prejudice.

Malevolence was identified as the most commonly reported attitude faced by the participants. It is unclear whether the frequency of shared malevolence experiences is due to it being experienced more than other expressions or if those experiences were more salient for participants. Regardless, experiences with malevolence appeared to resonate deeply with the participants and relate to damaging outcomes. The common link between malevolence and HCPs is consistent with broader research suggesting some HCPs believe patients with BPD are not truly unwell, manipulative, and deserving of less sympathy (for a review, see Ring and Lawn 2019). Research investigating intervention success on the four dimensions of prejudice towards mental health conditions in general has found no significant reductions in malevolence attitudes, and one study found a non‐significant increase following an education intervention (Kenny and Bizumic 2016; Richards et al. 2023). Further, Sheppard et al. (2023) found no relationship between increased contact and malevolence. This indicates that the traditional intervention methods (education and increased contact) may not be appropriate for reducing malevolence. The use of the PPBPD scale before and after educational interventions may support the development of tailored programs to address prejudice and determine change following the intervention.

This study further supports the need for lived experience perspectives in health research. Previous research using the PPBPD scale has focused on prejudice reported towards people with BPD among stigmatisers (Sheppard et al. 2023). In student and community samples, unpredictability and fear/avoidance were the highest reported prejudice, which may indicate a need to focus intervention efforts there first. Nevertheless, participants identified malevolence prejudice as the most prevalent for them. These experiences were linked to HCPs and family, and impacts such as reduced help‐seeking, shame, and decreased self‐esteem. Identifying the source and impact arguably holds greater importance than the reported level, as malevolence (even not very high) from a family member or source of care is likely to be far more damaging than higher levels of unpredictability or fear/avoidance attitudes from strangers. Lived experience perspectives in research can identify the most important sources and expressions of prejudice. This in turn can guide the development of interventions that can have the most positive impact for people living with BPD.

We acknowledge that researchers should involve people who have lived experience from the conception of research rather than later in the process. Nonetheless, we argue that developing the PPBPD model based on the PPMI model (Kenny et al. 2018) is a strength of this research. This model and scale were developed through thorough thematic and factor analysis of 27 existing measures of mental health stigma, which provided essential guidance in navigating this complex topic. Given the large body of literature the PPMI was built upon, we considered it likely that the main components of prejudice were already covered by this model and that any BPD‐specific aspects of prejudice could be integrated if identified in studies like this one. Therefore, we did not consider ground‐up development necessary, and this is supported by the experiences shared in this study.

### 5.1 Limitations

The small‐scale, convenience sample restricted the gender and cultural diversity of the interview participants. The participant pool was imbalanced, with only two male and two non‐binary people, which may bias the findings when considering non‐female perspectives. This is an ongoing limitation in BPD research as, despite data showing equal prevalence of BPD among males and females, research has often viewed BPD as a female‐predominant disorder (Qian et al. 2022). Qualitative research exploring help‐seeking and diagnosis experiences of men with BPD suggested that this gendered approach to BPD reinforces societal stigma towards and internalized stigma within men (Dean et al. 2024). Subsequently, male lived experiences may relate to the PPBPD model in ways that were not identified in this study and should be explored further. It is important that the applicability of the PPBPD model to the lived experience is investigated in other samples within and outside Australia.

Second, many participants came to the interviews with particular experiences and populations they wished to speak about, which may have become the focus of their interview. This does not decrease the value of their contributions; in fact, it highlights areas of great importance and potential primary intervention avenues. It is, however, important to acknowledge that a lifetime of experiences cannot be comprehensively shared in 90 min, and significant topics could have been missed. Further exploration of prejudice from the perspective of the stigmatized, both in the context of the PPBPD model and others, is recommended.

Further, the interview format may have influenced the results. Access to the questions prior to the interview may have primed participants to respond to the early open‐ended questions with the four factors of prejudice in mind. As a result, the interviews may have failed to identify additional prejudicial attitudes that could be integrated into the PPBPD model. Further research involving people with BPD may help to validate or expand the model.

## 6 Conclusion

This study provided validation for the PPBPD model from the lived experience perspective. It found that the four factors of Fear/Avoidance, Malevolence, Authoritarianism, and Unpredictability encompass attitudes of both support and prejudice experienced by people living with BPD. The PPBPD model and scale appear to be useful tools for identifying and understanding the connections between specific prejudice and its causes, sources, and impacts, which is vital to implementing effective, evidence‐based interventions.

## 7 Relevance for Clinical Practice

A model that is reflective of the lived experience, such as the PPBPD model, can be beneficial in guiding training and support for healthcare providers. Similar experiences with, and impacts of, negative attitudes from healthcare providers have been shared by participants in other studies (e.g., Carrotte et al. 2019; Vandyk et al. 2019). Linking malevolence prejudice to these experiences could inform support practices for healthcare providers and improve care and help‐seeking outcomes. For example, malevolence was reported to negatively relate to Davis's (1980) construct of empathic concern (Sheppard et al. 2023). Given that research shows that triage nurses with higher levels of self‐compassion also report higher empathy (Savieto et al. 2019), programs that encourage and support self‐compassion may prove useful. Such programs could be beneficial for healthcare providers and patients alike through improved healthcare provider‐client relationships and provision of care.

## Author Contributions

Hannah Sheppard led the conception and design of the study. She organized and conducted all interviews, transcribed and anonymised the transcripts, and completed the bulk of the analysis. Hannah also led the interpretation of the data, drafted the manuscript, submitted the manuscript, and agrees to be accountable for all aspects of the work in ensuring that questions related to the accuracy or integrity of any part of the work are appropriately investigated and resolved. Elizabeth Huxley contributed to the conception and design of the study, including the development of the interview questions. She acted as the on‐call registered psychologist in the event that any participant became distressed or requested a debrief upon interview completion. She contributed to the development of the codebook, conducted independent coding for the inter‐rater reliability analysis, contributed to the interpretation of the data, critically reviewed and edited draft versions of the manuscript, provided final approval of the version to be published, and agrees to be accountable for all aspects of the work in ensuring that questions related to the accuracy or integrity of any part of the work are appropriately investigated and resolved. Boris Bizumic supervised and contributed to the conception and design of the study. He contributed to the codebook development, analysis, interpretation of the data, critically reviewed and edited all draft versions of the manuscript, provided final approval of the version to be published, and agreed to be accountable for all aspects of the work in ensuring that questions related to the accuracy or integrity of any part of the work are appropriately investigated and resolved. Alison Calear contributed to the conception and design of the study. She contributed to the codebook development, analysis, interpretation of the data, critically reviewed and edited draft versions of the manuscript, provided final approval of the version to be published, and agrees to be accountable for all aspects of the work in ensuring that questions related to the accuracy or integrity of any part of the work are appropriately investigated and resolved.

## Funding

This work was supported by Mental Health Australia; National Health and Medical Research Council (Grant 1173146).

## Ethics Statement

This study was reviewed and approved by the Australian National University Human Research Ethics Committee (Protocol: 2021/397), and the procedures followed were in accordance with the Helsinki Declaration as revised in 2013. The participants provided their written informed consent to participate in this study.

## Conflicts of Interest

The authors declare no conflicts of interest.

## Supporting information


**Supinfo S1.** List of interview questions.

## Data Availability

Due to the study's approved ethics protocol, the data cannot be made freely available.
