# Supplementary material for: Is the Prejudice Towards People With Borderline Personality Disorder Model Reflective of Lived Experience?
Source: Int J Ment Health Nurs. 2026 Apr 17;35:e70258. doi: 10.1111/inm.70258 (PMC13088927; doi:10.1111/inm.70258)
Supplement: Supplementary file 1 — Supinfo S1. List of interview questions. [file INM-35-0-s001.docx]

# Supplemental Materials 1

**List of Interview Questions**

| Interview Section | Question |
| --- | --- |
| 1. General Questions | 1. What is your understanding of BPD? |
|  | 1. What do you think about people with BPD in general? |
|  | 1. How do you think other people perceive people with BPD? |
|  | 1. How do you feel about yourself and your diagnosis? |
|  | 1. Do you tell people in your life about your diagnosis? |
|  | 1. Have you had experiences where you felt people were prejudiced against you or your diagnosis? |
| 2. Experiences with Prejudice Dimensions | Brief introduction about previous research suggesting specific types of attitudes held towards people with BPD. |
|  | 1. Some participants expressed that they don’t think it would be easy to interact with someone who has BPD, and that they as a result prefer to avoid them, whereas other participants expressed that they were happy to interact with people with BPD and were very supportive of them. What have your experiences been with others? |
|  | 1. Many participants felt that society and people should be doing more to support people with BPD, while other participants expressed feelings of resentment or low sympathy: for example, the sentiment that people with BPD should support themselves and not expect handouts. What have your experiences been with others? |
|  | 1. Some participants expressed that treatment for the symptoms of BPD should always be the choice of the patient and never forced upon them, whereas other participants expressed the desire for society and professionals to control people with BPD, such as forcing the treatment on them, and not giving them sufficient control over their life. What have your experiences been with others? |
|  | 1. Lastly, some participants felt that the behaviour, emotions, interpersonal interactions of people with BPD are unpredictable, while other participants felt that their interactions with people with BPD would be just as predictable as someone without BPD. What have your experiences been with others? |
|  | 1. Do you think other groups of people (e.g., people with other psychological diagnoses or other social groups) face similar types of prejudice? |
| 3. Impact on the Individual | 1. How have these experiences affected you? |
| 4. PPBPD Framework Discussion | 1. Do these attitudes reflect your experiences? (*asked after the PPBDP framework was introduced, explained, and discussed*) |
| 5. Help-seeking | 1. When have you needed to seek help? |
|  | 1. What is your process of help seeking? Where do you seek help from? |
|  | 1. Are there any sources that you don’t use anymore? Do you mind if I ask why that is? |
| 6. Stigma Reduction | 1. Where do you think stigma reduction should be focused first? |
|  | 1. What would a reduction of stigma mean to you? |
|  | 1. What have you found most helpful? |
